# Comparing registered and resident populations in Primary Care Networks in England: an observational study

**DOI:** 10.3399/BJGPO.2022.0037

**Published:** 2022-10-05

**Authors:** Thomas Beaney, Gabriele Kerr, Benedict WJ Hayhoe, Azeem Majeed, Jonathan Clarke

**Affiliations:** 1 Department of Primary Care and Public Health, Imperial College London, London, UK; 2 National Institute for Health Research Applied Research Collaboration Northwest London, Imperial College, London, UK; 3 Department of Mathematics and Centre for Mathematics of Precision Healthcare, Imperial College London, London, UK

**Keywords:** service organisation, community care, practice organisation, primary health care, general practice, observational study

## Abstract

**Background:**

Primary Care Networks (PCNs) were established in England in 2019 and will play a key role in providing care at a neighbourhood level within integrated care systems (ICSs).

**Aim:**

To identify PCN ‘catchment’ areas and compare the overlap between registered and resident populations of PCNs.

**Design & setting:**

Observational study using publicly available data on the number of people within each Lower layer Super Output Area (LSOA) registered to each general practice in England in April 2021.

**Method:**

LSOAs were assigned to the PCN to which the majority of residents were registered. The PCN catchment population was defined as the total number of people resident in all LSOAs assigned to that PCN. The PCN catchment populations were compared with the population of people registered to a GP practice in each PCN.

**Results:**

In April 2021, 6506 GP practices were part of 1251 PCNs, with 56.1% of PCNs having 30 000–50 000 registered patients. There was a strong correlation (0.91) between the total registered population size and catchment population size. Significant variation was found in the percentage of residents in each LSOA registered to a GP practice within the same PCN catchment, and strong associations were found with both urban and rural status, and socioeconomic deprivation.

**Conclusion:**

There exists significant variation across England in the overlap between registered and resident (catchment) populations in PCNs, which may impact on integration of care in some areas. There was less overlap in urban and more deprived areas, which could exacerbate existing health inequalities.

## How this fits in

PCNs are expected to be a building block for ICSs but little is known about how they represent local communities. This study defines PCN catchment areas and identifies significant variation in the overlap between PCN registered patient populations and PCN catchment populations across the country. In urban and more deprived areas, fewer residents were registered to the same PCN catchment, which may impact on equity of access to care and integration of care in these areas.

## Introduction

PCNs are an organisational hierarchy in the NHS in England, introduced in the NHS *Long Term Plan* in 2019.^1^ The intention was to boost the capacity of primary care to work at scale, with PCNs representing groups of individual GP practices with combined registered list sizes, typically between 30 000 and 50 000 patients.^1^ Significant funding incentives are attached to the formation of PCNs, including for the employment of additional primary care staff, such as social prescribers, pharmacists, and physiotherapists. Previous work has identified the rapid uptake of PCNs, with 6758 GP practices forming 1250 PCNs by January 2020.^2,3^ PCNs are expected to have a wide remit, beyond the employment of additional multidisciplinary team members.^4^ PCNs are also expected to form part of an ICS, and engage in population health management, as well as becoming anchor institutions for community services.^5,6^

The concept of ‘place’ is fundamental to the formation of ICSs, which are designed to provide services across a geographical footprint, in many cases aligned with existing local authority boundaries.^7^ Within ICSs, PCNs are anticipated to be one of the building blocks for integrating care at a ‘neighbourhood’ level. However, GP practices in England receive funding and provide care for patients who are registered, rather than those resident within a fixed geographical area. Although GP practices have defined practice boundaries, these often overlap with other practices and span multiple clinical commissioning groups (CCGs); practices may also register patients ‘out-of-area’.^8,9^ As a result, practices’ registered patients may be widely dispersed and, given that near practices may not belong to the same PCN, understanding the neighbourhood in which patients are likely to live and their relationships with other community services and secondary care providers is complex. This may result in some practices, and by extension PCNs, needing to work with multiple different providers to provide care, rather than integrating with a single provider organisation. Consequently, there is a pressing need to understand how reflective GP practice registered populations are of the people resident in their neighbourhoods and whether differences exist according to geography.

There is growing interest for NHS organisations to understand their local populations through defining ‘catchment’ areas.^10–12^ Catchments can be viewed as the geographic area in which patients attending an organisation are likely to live. Various approaches exist to defining catchments, with two common approaches assigning small unit geographies to the provider to which either a pre-defined proportion of residents are registered, or where the majority of residents are registered.^10,12–14^ In this article, the populations of PCNs in England are described by (i) registered patients and (ii) catchment areas, defining a catchment as the area for which a PCN is the dominant primary care provider. The overlap between the populations registered to practices within a PCN and the populations resident within PCN catchment areas defined in the study are then compared, and the variation in the coverage of PCNs across England is thereby characterised.

## Method

### Data sources

This study used publicly available data from NHS Digital on the number of patients registered at each GP practice in England living in each Lower layer Super Output area (LSOA) in England in April 2021.^15^ An LSOA is a small-unit geographic area defined by the Office for National Statistics, representing on average approximately 1700 people, with 32 844 LSOAs in England.^16^ GP practice PCN and CCG allocations were sourced from NHS Digital as of May 2021.^17^ May 2021 assignments were chosen in preference to April 2021 assignments owing to a change in CCG boundaries in the May release.

### Defining a PCN catchment

**Figure 1.**
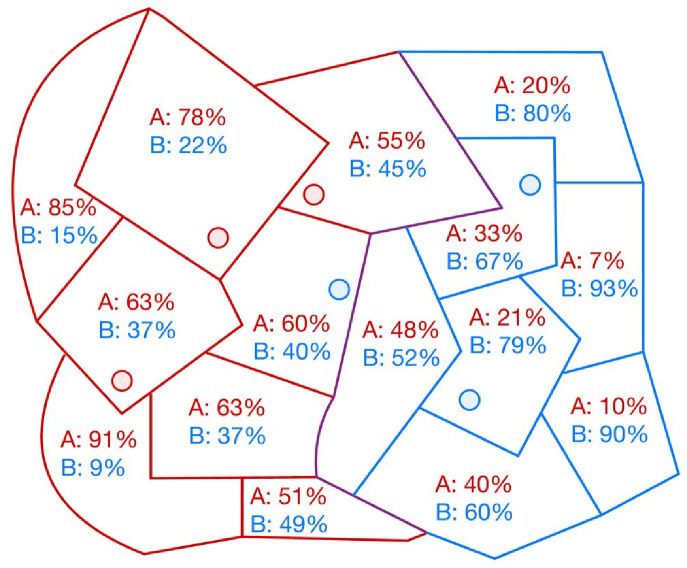
Schematic representation of the allocation of Lower layer Super Output Areas (LSOAs) to primary care networks (PCNs). Each circle represents a GP practice, coloured according to its PCN (red = PCN A, blue = PCN B). Each polygon represents an LSOA. The percentages in each polygon show the percentage of residents in the LSOA who are registered to practices forming part of PCN A and PCN B. The boundaries of LSOAs are coloured according to the colour of the PCN to which they are assigned by the first-past-the-post (FPTP) method

For each LSOA, the proportion of patients registered at practices within each PCN was calculated. A first-past-the-post (FPTP) method was used to assign an LSOA to the PCN to which the majority of LSOA residents were registered (Figure 1). This approach was chosen to ensure each LSOA was assigned to a single PCN, unlike alternative methods that can result in non-assignment or multiple assignment of LSOAs to PCNs.^10,12^ The PCN catchment was defined as the set of all LSOAs assigned to the PCN.^10^ In a total of six (0.02%) LSOAs, there was a tie between two majority providers, and the LSOA was assigned randomly to a PCN to avoid double-counting of the LSOA. All residents of each LSOA assigned to the PCN catchment were counted towards the total catchment population. The 118 GP practices that were not part of a PCN were removed for the purposes of defining a catchment area and their populations removed for calculating denominator populations.

### Statistical analysis

For each PCN, the registered population was defined ( $PCN_{registered}$ ) for each PCN as the sum of all patients registered to GP practices that were part of the PCN:


(1) $$PCN_{registered}=\;\sum_{i=1}^{N}GP_{i}$$

Where ${GP}_{i}$ is the population registered to GP practice *i,* and *N* is the total number of GP practices in the PCN. For each PCN, the catchment population was also defined ( ${PCN}_{catchment})$ ) as the sum of all residents in each LSOA assigned to the PCN:


(2) $$PCN_{catchment}=\;\sum_{j=1}^{M}LSOA_{j}$$

Where ${LSOA}_{j}$ is the population resident in LSOA *j,* and *M* is the total number of LSOAs in the PCN catchment.

The following two metrics were calculated for each PCN: (i) the percentage of the catchment population who were registered to the same PCN ( percentage registered ); and (ii) the percentage of the registered population who were resident in the PCN catchment ( percentage resident ):

(3) $$PCN\;percentage\;registered=\frac{|\;A\;\cap \;B\;|}{|\;B\;|}\times 100$$

(4) $$PCN\;percentage\;resident=\frac{|\;A\;\cap \;B\;|}{|\;A\;|}\times 100$$

Where *A* represents people in the PCN registered population, *B* represents people in the PCN catchment population and *A*∩ *B* represents people in both the PCN registered population and the PCN catchment population.

For each LSOA, the percentage of the LSOA resident population who were registered to a GP practice in the PCN catchment to which the LSOA was assigned was calculated:

(5) $$LSOA\;percentage\;resident=\frac{|\;A\;\cap \;C\;|}{|\;C\;|}\times 100$$

Where *C* represents people resident in the LSOA and *A* ∩ *C* represents people resident in the LSOA who are also in the PCN registered population.

A z-test was used to measure the association between the proportion of patients in the LSOA registered to the PCN in urban versus rural LSOAs, and by quintiles of Index of Multiple Deprivation (IMD).^18,19^ Categories A1, B1, C1, and C2 were defined as urban, and D1, D2, E1, and E2 were defined as rural.^18^ Analyses by IMD were also stratified by urban versus rural status and the proportion of patients in the LSOA registered to the PCN compared between IMD quintiles using ANOVA with post-hoc Tukey’s honest significance test. All analyses were conducted using R (version 4.0.2). PCN catchments were mapped using the R package tmap.

## Results

### PCN registered populations

In April 2021, 60 744 002 patients were registered to 6624 GP practices in England. A total of 118 (1.8%) practices were not recorded by NHS Digital as being allocated to a PCN and were distributed among 44 CCGs, with a maximum number of 11 per CCG. The remaining 6506 (98.2%) practices were included in the study, which were part of 1251 PCNs, representing 60 212 490 (99.1%) of all registered patients. The number of practices per PCN ranged from 1–31, with a median of five practices (interquartile range [IQR] 3–6). Also, 99.1% of PCNs include GP practices from only a single CCG, and 0.9% include practices from two CCGs, with no PCNs including practices from more than two CCGs. The number of patients registered to practices within each PCN ranged from 14 248–257, 931, with a median of 44 151 patients (IQR 35 811–54 699; Supplementary Figure 1A). It was found that 56.1% of PCNs had between 30 000 and 50 000 registered patients, 8.2% of PCNs had <30 000 registered patients, and 35.7% of PCNs had >50 000 registered patients.

### PCN catchment populations

**Figure 2.**
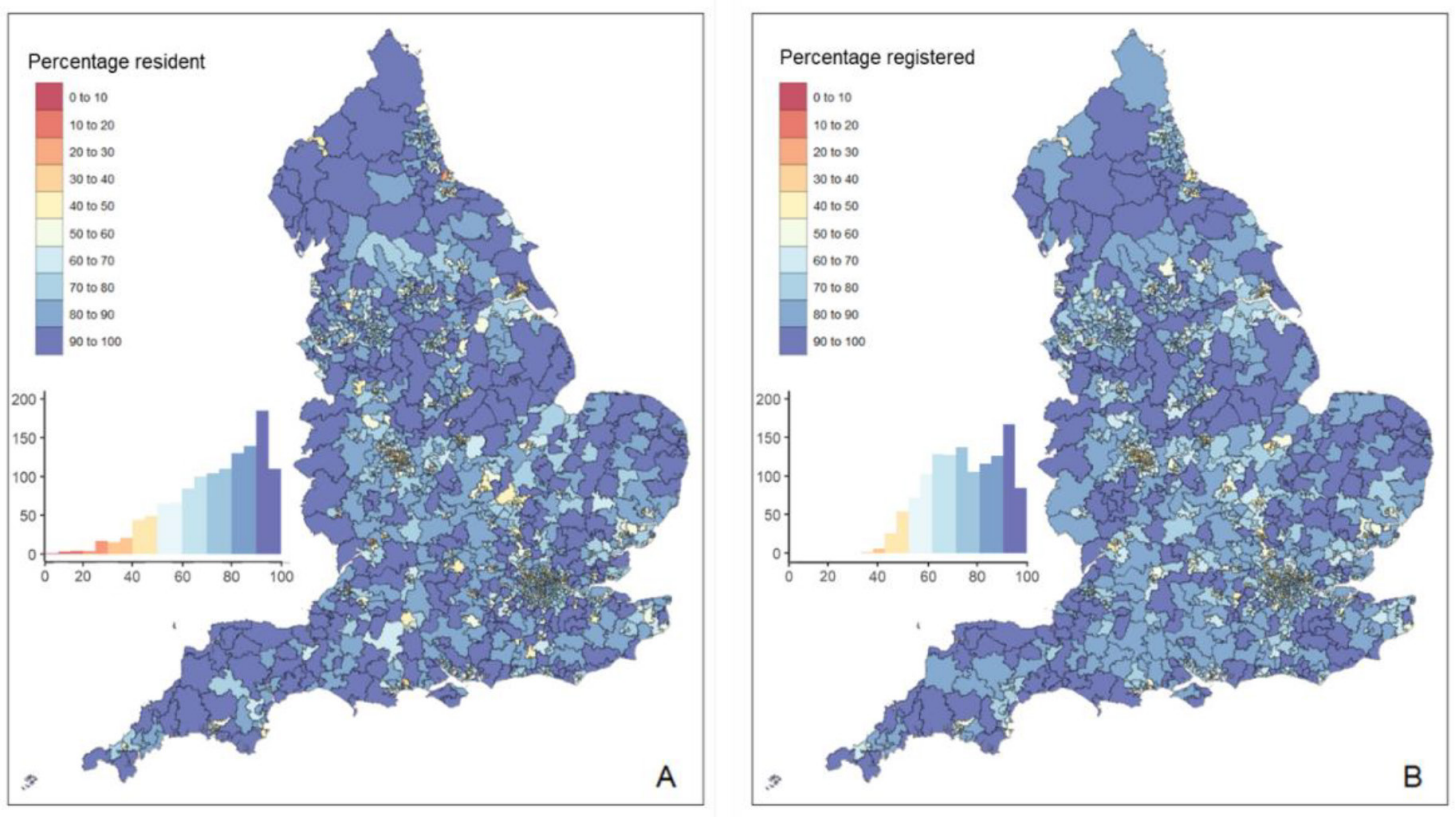
Primary care network (PCN) populations: panel A shows the percentage of the PCN registered population who are resident in the catchment of the PCN; panel B shows the percentage of each PCN catchment population who are registered to a GP practice within the PCN

LSOAs were assigned to the PCN to which the majority of residents were registered using the FPTP method. All PCNs were assigned at least one LSOA. The size of each PCN catchment ranged from 2–150 LSOAs, with a median of 24 LSOAs per PCN (IQR 19–31). The resulting PCN catchments are presented in Figure 2 and in the online interactive map (https://healthnetworks.github.io/projects/primary_care_networks.html). PCN catchment populations ranged from 6916–254 724, with a median of 44 718 patients (IQR 34 808–56 725; Supplementary Figure 1B).

47.9% of PCNs had a geographic catchment of 30 000–50 000 patients with 38.4% having a geographic catchment of >50 000; and 13.7% of PCNs had a geographic catchment of <30 000 patients.

### Comparison of PCN registered populations and catchment populations

**Figure 3.**
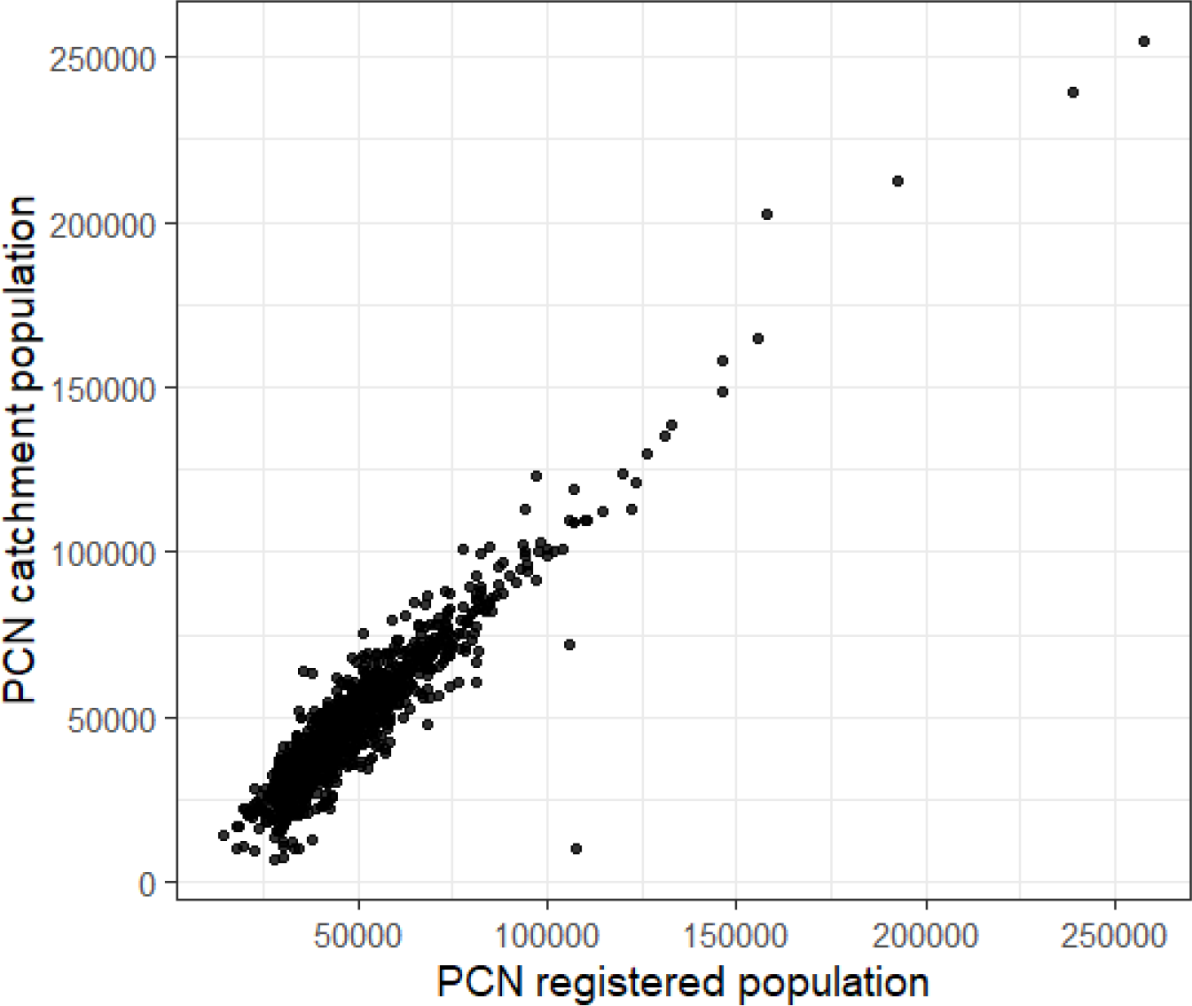
Total PCN catchment population against total PCN registered population for all PCNs in England

There was strong correlation between the total PCN registered population and catchment population sizes for each PCN (*P* = 0.91, *P*<0.0001) (Figure 3). The absolute difference in population size between registered population and catchment population for each PCN ranged from 9–97 772 patients with a median (IQR) of 3432 (1432–6819). The largest difference was for Babylon GP At Hand PCN, which had a registered population of 107 726 and a catchment population of 9954.

The percentage of people in the PCN registered population who were also resident in the catchment of the same PCN ('percentage resident') ranged from 4.0–99.3% with a median of 77.4% (IQR 61.4–89.3). The percentage of people in the PCN catchment population who were also registered to the PCN ('percentage registered') ranged from 33.1–99.6%, with a median of 73.8% (IQR 61.9–87.7). (Figure 3). Excluding Babylon GP at Hand PCN, the median 'percentage resident' was 77.4% (range 10.2–99.3%, IQR 61.5–89.3) and the median 'percentage registered' 73.9% (range 33.1%–99.6%, IQR 62.0–87.7).

### Variation in PCN registration by LSOA

**Figure 4.**
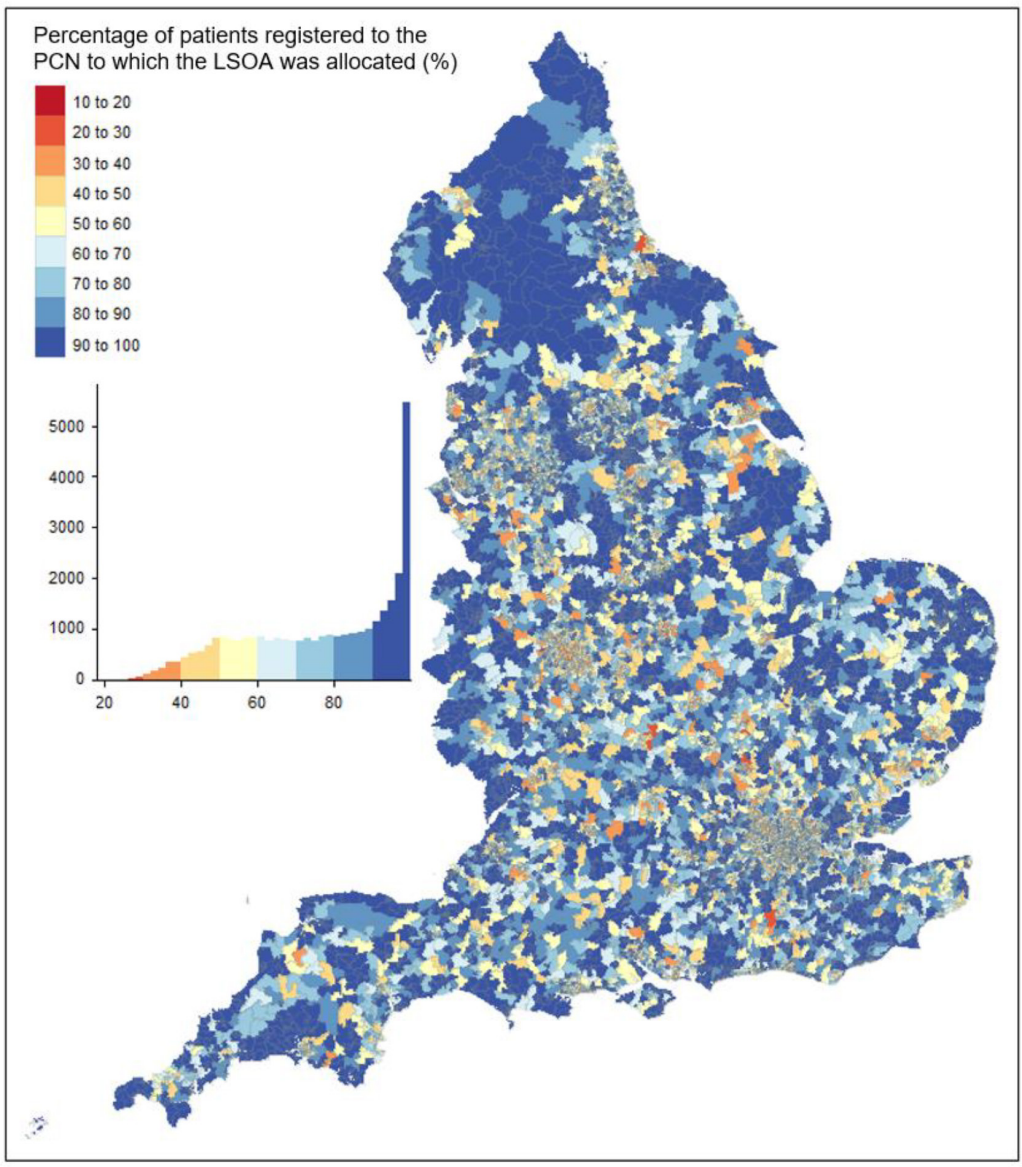
Percentage of residents within each Lower layer Super Output Area (LSOA) registered to a GP practice in the primary care network (PCN) catchment to which the LSOA was assigned

The number of PCNs to which LSOA residents were registered ranged from 1–89, with a median of eight PCNs per LSOA (IQR 6–13). The percentage of residents in each LSOA who were registered to a GP practice in the PCN catchment ranged from 19.5–100.0%, with a median of 79.6% (IQR 59.4–95.2) There was significant variation by geography, with lower percentages apparent in more urban areas (Figure 4).

Relative to rural LSOAs, urban LSOAs had on average 10.7% (95% confidence interval [CI] = 10.1 to 11.2; *P*<0.0001) fewer patients who were registered in the PCN catchment. There was a strong association with deprivation (*P*<0.0001) with LSOAs in the most deprived quintile having on average 9.4% (95% CI = 8.7 to 10.0; *P*<0.0001) fewer patients who were registered in the PCN catchment, compared with LSOAs in the least deprived quintile.

In an analysis stratified by urban or rural status, there remained a strong association with deprivation in urban (*P*<0.0001) and rural LSOAs (*P*<0.0001). In urban areas, LSOAs in the most deprived quintile had an average of 8.2% fewer residents who were registered in the PCN catchment than those in the least deprived quintile (95% CI = 7.3 to 9.3%; *P*<0.0001; Table 1). In rural areas, there was a trend towards lower proportions registered on moving from the IMD quintile 1 (most deprived: 89.1%) to IMD quintile 4 (83.4%), but with a higher percentage in IMD quintile 5 (87.3%).

**Table 1.** Mean percentage of residents in each Lower layer Super Output Area (LSOA) registered to a GP practice within the primary are network (PCN) catchment by urban or rural status and deprivation quintile

Deprivation quintile	Urban		Rural	
	Estimate	95% CI	Estimate	95% CI
1 (most deprived)	71.3	70.8 to 71.8	89.1	87.1 to 91.2
2	71.9	71.4 to 72.4	86.1	84.9 to 87.3
3	73.6	73.0 to 74.1	83.4	82.5 to 84.3
4	75.9	75.3 to 76.4	83.4	82.5 to 84.2
5 (least deprived)	79.5	79.0 to 80.1	87.3	86.5 to 88.1

## Discussion

### Summary

Two years after the introduction of PCNs, most GP practices in England are part of a PCN. There is a wide range in the registered population size of PCNs, with only 56.1% in the 30 000–50 000 range originally recommended by NHS England. Assigning geographically defined PCN catchment areas resulted in similar average population sizes, but there was large variation across England in the overlap between the registered and resident catchment populations. In one-quarter of areas, <60% of people registered to a GP practice in the PCN were resident within the PCN catchment area, while in others this approached 100%. LSOAs in urban areas tended to have less overlap between registered and resident populations compared with those in rural areas, as did LSOAs in more deprived versus less deprived areas. However, within urban and rural areas, there were differences in the associations with deprivation, with lower percentages registered in more deprived compared with less deprived urban areas, but a trend towards higher percentages registered in more deprived areas. This may in part reflect differing patterns of mobility in rural and urban areas across the socioeconomic gradient.

### Strengths and limitations

A strength of the work is the use of data covering all patient registrations to GPs in England, stratified by every LSOA, allowing the comparison of both registration and residence patterns. A limitation of the data is the inclusion only of patients who are registered to a GP practice. Some vulnerable patient groups, such as Travellers and people experiencing homelessness, are less likely to be registered with GPs and so may not be represented by the findings.^20^ Second, the LSOA of residence of a patient is derived from their address as registered with their GP, and therefore may not be up to date. If some patients move further from their practice and do not re-register or update their address, the concordance observed in this study could be expected to be an overestimate of the true agreement between primary care registration and residence. The aim of the study was to compare registration patterns across the country, and according to urban and rural status and socioeconomic deprivation, but not to measure the causal effects of these factors on registrations. The associations seen in this study will be at least partially explained by sociodemographic factors, which impact on patient preference for proximity to and choice of GP practice; for example, if younger people are less likely to update their address than older people after moving, or those in employment choose to register at a location close to their place of work rather than residence, consistent with a previous study finding higher over-registration in London than other parts of England.^21^

### Comparison with existing literature

The size of PCNs based on registered patients are similar to a previous study using data from January 2020, which identified a similar number of PCNs (*n* = 1250) and median size, indicating little change in the intervening period up to April 2021.^2^ This study also found that 40% of PCNs were outside of the recommended size range. A second study using a data-driven approach to defining PCN catchment areas in London also suggested that larger PCN size may be more reflective of underlying patient registration patterns.^14^

To the authors' knowledge, this is the first study that has defined a geographical catchment for PCNs. However, geographical catchments have been described in secondary care settings and different methods have been proposed. The FPTP method was chosen as it results in the assignment of every LSOA to one and only one PCN; other approaches, such as a proportional flow method may assign an LSOAs to more than one catchment or leave others unassigned.^12^

### Implications for research and practice

With the formation of ICSs, which have well-defined geographical footprints, there is a need to understand how PCNs will fit into local ICS ‘neighbourhoods’.^7,22^ This work highlights that around one-quarter of people registered to a GP practice in a PCN are not living within the nominal catchment area of their PCN, but also that there is significant variation nationally, meaning that some PCNs may be differently able to achieve the objective of providing care to a geographic community of patients. In some parts of the country, most patients live outside the bounds of the geographic catchment of their registered PCN. Instead, these PCN communities may be imagined as overlapping extensively with one another and without a discrete geographic identity. In such areas, the challenge of accountability of individual PCNs and collaboration with local place-based services is likely to be greater than in areas with little overlap. Identifying patterns of overlap at a local level may inform strategies for collaboration both between neighbouring PCNs and individual GP practices within PCNs where overlap is particularly great.

The finding that PCNs in urban areas, and particularly those in more deprived urban areas, tend to have less overlap between registered and catchment populations than those in rural, and less deprived areas, highlights the possibility of inequities in care provision. This indicates a need to consider local context, including population movement and registration patterns in policy decisions and commissioning of integrated care services. These findings also suggest that in urban areas, and more socioeconomically deprived urban areas in particular, the implementation of larger PCNs consisting of more practices may facilitate better matching between the registered patients of a PCN and its catchment residents.

With increasing use of remote consultations during the COVID-19 pandemic, further research is needed to understand patient and practice priorities around the importance of proximity to their GP practice, and whether preferences have changed over time. The Babylon GP At Hand PCN, for example, was providing a significant number of remote consultations before the pandemic^23,24^ and was found in the present study to have a much larger registered population than catchment population, indicating a wide geography over which patients are registered. With a move to greater digital health care during the pandemic, if GP practices register more patients ‘out-of-area’, a similar pattern may be replicated across other PCNs over time and become a hurdle to further integration of care. Some healthcare services, such as mental health teams and district nursing, may only accept patients resident within a defined geography, and so a widely dispersed patient population will increase the number of organisations a GP practice within a PCN needs to work with, potentially resulting in higher workloads, inhibiting organisational continuity of care and integration of services, and ultimately impacting on patient care.

In conclusion, as of April 2021, most GP practices in England have formed part of a PCN. The study finds substantial variation across the country in the overlap between people and place in PCNs. Lower overlap was more common in urban and more deprived areas, which is likely to exacerbate existing health inequalities. Low overlap in some areas will significantly hamper the ability of PCNs to integrate with community services and ICSs, and with greater use of remote consultations during the COVID-19 pandemic, there is a risk that this overlap erodes over time.

## Data

This study used publicly available data from NHS Digital.
